# Effects of the COVID-19 pandemic on general health and malaria control in Ghana: a qualitative study with mothers and health care professionals

**DOI:** 10.1186/s12936-023-04513-6

**Published:** 2023-03-06

**Authors:** Anna-Katharina Heuschen, Alhassan Abdul-Mumin, Abdulai Abubakari, Faith Agbozo, Guangyu Lu, Albrecht Jahn, Olaf Müller

**Affiliations:** 1 Institute for Global Health, University Hospital Heidelberg, Ruprecht-Karls-University, Heidelberg, Germany; 2 School of Medicine, Department of Paediatrics and Child Health, University for Development Studies, Tamale, Ghana; 3 Tamale Teaching Hospital, Tamale, Ghana; 4 School of Public Health, Department of Global Health, University for Development Studies, Tamale, Ghana; 5 Fred Binka School of Public Health, Department of Family and Community Health, University of Health and Allied Sciences, Hohoe, Ghana; 6 School of Public Health, Medical School, Yangzhou University, Yangzhou, China

**Keywords:** Malaria, Health services, COVID-19, Children under 5, Mothers, Ghana, Sub-Saharan Africa, SSA

## Abstract

**Background:**

COVID-19 has severely impacted health systems and the management of non-COVID-19 diseases, including malaria, globally. The pandemic has hit sub-Saharan Africa less than expected; even considering large underreporting, the direct COVID-19 burden was minor compared to the Global North. However, the indirect effects of the pandemic, e.g. on socio-economic inequality and health care systems, may have been more disruptive. Following a quantitative analysis from northern Ghana, which showed significant reductions in overall outpatient department visits and malaria cases during the first year of COVID-19, this qualitative study aims to provide further explanations to those quantitative findings.

**Methods:**

In the Northern Region of Ghana, 72 participants, consisting of 18 health care professionals (HCPs) and 54 mothers of children under the age of five, were recruited in urban and rural districts. Data were collected using focus group discussions with mothers and through key informant interviews with HCPs.

**Results:**

Three main themes occurred. The first theme—general effects of the pandemic—includes impacts on finances, food security, health service provision as well as education and hygiene. Many women lost their jobs, which increased their dependance on males, children had to drop out of school, and families had to cope with food shortages and were considering migration. HCPs had problems reaching the communities, suffered stigmatisation and were often barely protected against the virus. The second theme—effects on health-seeking—includes fear of infection, lack of COVID-19 testing capacities, and reduced access to clinics and treatment. The third theme—effects on malaria—includes disruptions of malaria preventive measures. Clinical discrimination between malaria and COVID-19 symptoms was difficult and HCPs observed increases in severe malaria cases in health facilities due to late reporting.

**Conclusion:**

The COVID-19 pandemic has had large collateral impacts on mothers, children and HCPs. In addition to overall negative effects on families and communities, access to and quality of health services was severely impaired, including serious implications on malaria. This crisis has highlighted weaknesses of health care systems globally, including the malaria situation; a holistic analysis of the direct and indirect effects of this pandemic and an adapted strengthening of health care systems is essential to be prepared for the future.

## Background

The global spread of SARS-CoV-2, causing coronavirus disease 2019 (COVID-19), arrived on the African continent on February 14, 2020, with a first case reported from Egypt [1]. The World Health Organization (WHO) declared COVID-19 a pandemic on March 11 [2]; 1 day later, Ghana reported its first case [3]. Less than 3% of all COVID-19 cases and deaths were reported by African countries during the peak of the pandemic in 2020 and 2021, while the continent is home to 17% of the world’s population [4]. Besides a much younger population, different climate conditions and potentially protective immunological interactions, underreporting seems to be the main explanation of this trend; a recent study estimated that in sub-Saharan Africa (SSA), only 1.4% of all COVID-19 infections and 35% of all COVID-19 deaths have been reported [5].

In addition to the direct health impacts, the indirect effects of COVID-19 have challenged communities and health systems globally. The public health measures taken and other repercussions to the pandemic have led to numerous socio-economic impacts, worsening the social inequality and threatening non-COVID-19 health programmes, e.g. malaria control activities [6]. These aspects strongly affect SSA, as many structures have already been fragile prior to the onset of the pandemic. During the 2014–2016 West African Ebola outbreak, for example, less outpatient department (OPD) visits and malaria cases were reported from health facilities, while it is estimated that the excess malaria deaths, resulting from indirect effects of the epidemic on malaria health services, exceed the direct Ebola deaths [7]. These experiences have led to numerous pessimistic modelling studies regarding the impact of COVID-19 on malaria during the first months of 2020, predicting up to a doubling of malaria mortality, especially resulting from reduced access to anti-malarial drugs and reduced insecticide-treated nets (ITNs) distribution [8, 9]. The progress made in the fight against malaria since the start of this century had already slowed down in the last 5 years before the pandemic and risked being reversed completely by adverse effects of COVID-19 [10].

The 2021 WHO World Malaria Report states that there have been 6% more malaria cases and 12% more malaria-related deaths globally in 2020 compared to 2019, 68% of those having been attributed to the pandemic, and over 95% of these increases are attributed to SSA [10]. In 2021, the case incidence remained similar to 2020, while the mortality rate increased in 2020 compared to 2019, but decreased again slightly in 2021 (14 in 2019, 15.1 in 2020, 14.8 in 2021); these trends are suggesting that the countries’ efforts averted worst-case scenarios [11]. In absolute numbers, disruptions due to the COVID-19 pandemic have led to additional 13.4 million malaria cases and 63,000 malaria deaths in 2020 and 2021 [11]. Some statistics from malaria-endemic countries revealed diverse effects on malaria morbidity and mortality; in high malaria-endemic countries, fewer cases were seen in health facilities while in low malaria-endemic countries cases increased [12–19]. For northern Ghana, a retrospective analysis of quantitative data from April to September 2020 compared to the pre-COVID-19 period from 2015 to 2019 found a 26% decrease in overall OPD malaria cases with even stronger reductions in children under the age of five and inpatients (up to 67%) [20]. Similar findings were seen in other regions of Ghana, as investigated in a qualitative study in a rural community in the Eastern Region [21] and in a quantitative study in the Volta Region [22]. Verner et al. [22] further reported an increase in cerebral malaria, resulting in shorter admission durations and followed by a higher malaria related mortality in 2020, compared to the years 2016–2019. The objective of this qualitative study is to provide further explanations to those trends by investigating the perspectives of mothers and health care professionals (HCPs) on how COVID-19 has impacted their lives, health services and the malaria situation.

## Methods

### Study design and period

This study is a descriptive qualitative inquiry in the context of an explanatory mixed methods approach. The qualitative research follows a scoping review [23] and a quantitative data analysis [20] and aims to add explanations and insights from the field to those findings. Key informant interviews (KIIs) with health care professionals and focus group discussions (FGDs) with mothers of children aged 6 months to 5 years were conducted in a rural and an urban setting during April and May 2022, shortly after most of Ghana’s COVID-19 control measures and restrictions were relieved.

### Study setting

The data collection took place in two districts of the Northern Region of Ghana. Ghana has a population of 31 million with the Northern Region having a population of 2, 3 million in 2021 [24]. The socio-economic situation of that region is below the average of the country: it has the lowest rate of primary school attendance (59%), the lowest female literacy rate (44% among women aged 15–24 years) and the highest rate of under-five mortality (124/1000 live births) [25]. Tamale Metropolis—which is the capital city of the Northern Region—is mainly an urban district. Kumbungu District is mainly rural and is located 30 km away from Tamale. The city of Tamale has 374,744 inhabitants, Kumbungu District has a population of 110,586 [24]. For both districts, two primary health care facilities (community health centres) were included in the study. Further, the Tamale Teaching Hospital, a tertiary health facility located within the Tamale Metropolis and serving the entire northern belt of Ghana, was a third study site. Ghana has instituted a national health insurance scheme since 2003, covering many common disease conditions, including malaria, and the costs of many medications, including anti-malarials [26]. In 2021, national coverage of the insurance scheme was at 54%, but the coverage for the Northern Region was only 40% [27]. Malaria was the main reason for OPD visits in 2020 and 2021 and responsible for about 20% of all health facility visits of the country [27].

### Study participants and sampling

**Fig. 1:**
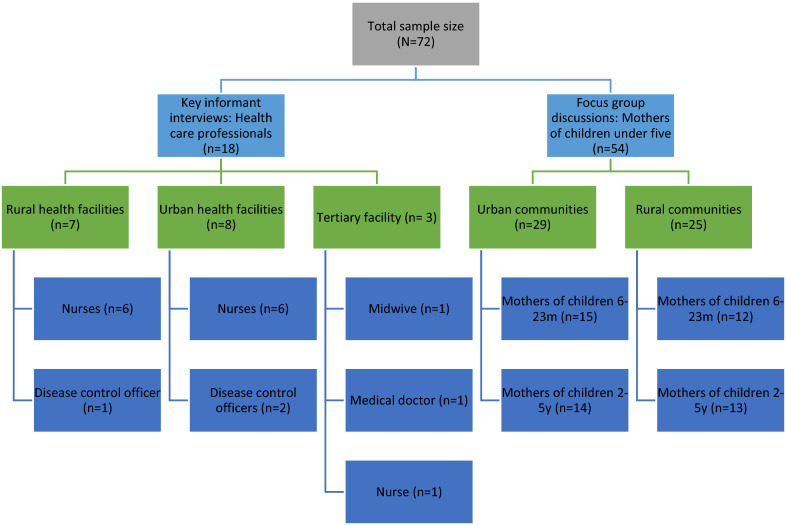
Study participants, categorised between mothers and health care professionals, distributed by study setting

Figure 1 shows the distribution of the number of HCPs and mothers recruited, as well as the respective study setting classified into rural and urban. Overall, 72 adults (≥ 18 years) participated in this study. The FGDs were conducted with mothers of children aged 6–23 months (pregnant during the first and second wave of the COVID-19 pandemic in Ghana from April to August 2020, and from January to April 2021 [28]) and mothers of children aged 2–5 years (that were infants during the peak of the COVID-19 pandemic). The districts and communities were chosen purposively to provide a more diverse, rural–urban perspective on the study objective while the four primary health care centres in these districts were chosen randomly. 18 KIIs were conducted with HCPs from different professional groups and hierarchical levels, consisting of three from a tertiary facility, seven from a rural and eight from an urban health centre. The categories included nurses (n = 13), disease control officers (n = 3), a medical doctor (n = 1) and a midwife (n = 1). The interviews took place in a calm room in the respective workplaces of the health professionals with no external persons listening and no need to travel. The study team also informed community volunteers about the study process and topic and asked them to recruit an in advance fixed number of eligible mothers who had children under 5 years of the respective age categories regarding their willingness to participate. The mothers were briefed by the community volunteers about the study topic and the interview style. 54 who were interested to participate, showed up on the scheduled day. They were given further explanations by the study team and were interviewed after giving their consent. 25/54 of them (13/25 mothers of children aged 2–5 years, 12/25 mothers of children aged 6–23 months) lived in a rural community, 29/54 in an urban setting (14/29 mothers of children aged 2–5 years, 15/29 mothers of children aged 6–23 months). The FGDs were held in a calm place in the communities of the mothers (in a summer hut/ under a large tree) with no need to travel and with no external community members listening, respecting the privacy of the participants.

### Data collection

Based on a prior scoping review [23] and a quantitative data analysis of routine surveillance data on malaria [20], interview guides were developed and reviewed by the research team. The four FGDs with mothers (n = 27) of children aged 6–23 months focused on the effects of COVID-19 during pregnancy, while the four FGDs with mothers of children aged 2–5 years (n = 27) focused on the effects of COVID-19 on children. The interviews with the HCPs focused on the effects of COVID-19 on specific aspects of their work. One day was used to train two local research assistants, both public health students who had experiences as research assistants in other projects. The interview and FGD guides were translated into the predominantly spoken local language at the study area (*Dagbanli*). Data was collected by the principal investigator and the research assistants. The KIIs with HCPs were conducted by the principal investigator and research assistants in the respective health facilities under face masks protection as one-to-one conversations in English and tape recorded. The FGDs with mothers were held outdoors, one research assistant moderated, one took notes about the content and the principal investigator took notes about the behaviour and tape recorded. All participants were anonymized after having filled the consent form.

### Data management and data analysis

The audio recordings of the FGDs were translated into English, transcribed and deleted after transcription. The transcripts were read and analysed independently by two team members using Nvivo 12. Codes were created inductively by reading the raw textual data. The key textual segments that highlighted the concepts related to COVID-19 and malaria epidemiology were identified and labelled using the word cloud function to depict the frequency and importance attached to the concept. The textual data was coded deductively using predefined concept-driven codes identified from literature search. After the initial open coding process, the first level codes established by inductive and deductive content analysis were aggregated into second level categories by assessing interconnections between code categories and by structuring those into broader themes. Queries to extract portions of the transcript were done by highlighting the notes alongside using the coding matrix and simple crosstab function to compare sub-categories. Data analysis was structured around the three main themes that emerged: effects of the COVID-19 pandemic on everyday life, effects on general health care and health-seeking, and effects on the malaria situation.

### Ethical approval

Ethical approval was obtained from the Navrongo Health Research Centre Institutional Review Board (ID: NHRCIRB437) and the ethical commission of the University Hospital Heidelberg (S-308/2022). Additionally, the respective district, community and health centre/hospital in-charges were asked for and provided their permission.

Written informed consent was obtained from every study participant: the mothers, consisting of an important illiterate fraction, received oral information in their local language and were then asked to sign by fingerprint if they agree in participation, an independent witness was present during this step. The HCPs, all literate and with English language skills, got a written information sheet in English before being asked to sign.

## Results

### Socio-demographic data

Employment of mothers in the urban areas included vendors, seamstresses and housewives, in decreasing order; whereas mothers in the rural areas were vendors, farmers, housewives, shea butter producers and seamstresses, again in decreasing order.

Three main themes emerged during the interviews with mothers and HCPs: (1) General effects of the COVID-19 pandemic, (2) effects of COVID-19 on health-seeking and health services, and (3) effects of the pandemic on the malaria situation. These main themes have been grouped into 12 sub-themes (Tables 1, 2, 3). The rural–urban perspectives have not been included in the tables as the differences were mostly insignificant. When noteworthy differences occurred, comments have been included in the continuous text.

**Table 1:** General effects of the COVID-19 pandemic

	Health care professionals	Mothers
Finances, livelihood and food security	Change in workload Stigmatization	Loss of income Over-dependence on male reference persons High costs of living Dropout of school and further education (children & mothers)
Health service provision	Staff shortage Lack of PPE Limited outreach to rural communities Reduced health care funding	Limited accessible health services Inability to buy medications, PPE, pay for transportation
Education and hygiene	Misinformation about COVID-19 Increased health education about COVID-19 Impaired control and information on non-COVID-19 diseases	Improved personal & environmental hygiene Inadequate education on non-COVID-19 diseases

*PPE* personal protective equipment

**Table 2:** Effects of the COVID-19 pandemic on health-seeking

	Health care professionals	Mothers
Fear of COVID-19 infection	Reduced patient contact/handling Low patient load for routine services, only for emergencies	Fear of visiting health facilities and schools Fear of non-COVID-19 illnesses Less attendance for ANC, increased home deliveries, use of traditional birth attendants
COVID-19 testing	Lack of testing	Stigmatisation for COVID-19 like symptoms Fear and stigma of testing positive and isolation
Behaviour of health care providers	Poor quality care in symptomatic patients Patients’ fear of infection from HCPs Stigmatization	Low HCPs accessibility Low quality of HCPs care in symptomatic patients Disruption of ANC and community outreach clinics
Access to clinics and HCPs	Less routine child vaccinations ANC closure in tertiary facility	HCPs shortage Health facility closures/ reduced opening hours/ reduced emergency services at night
Access to medicines	Varied medicine availability: low demand leading to no effect/ reduced stock from supply issues	Lack of medicines High medication prices Self-medication using over-the-counter drugs preferred to herbal treatment

*ANC* antenatal care

**Table 3:** Effects of the COVID-19 pandemic on the malaria situation

	Health care professionals	Mothers
Malaria preventive measures	Inconsistent chemoprophylaxis intervals Reduced access to chemoprophylaxis and ITNs Low malaria funding	Lack of chemoprophylaxis and ITNs Non-willingness to take chemoprophylaxis
Differential diagnosis of malaria and COVID-19	Inadequate education on symptomatic similarities Increased malaria testing leading to shortages of malaria test kits Increase in severe and complicated malaria in health facilities Checklist-based COVID-19 diagnosis; COVID-19 test lacking Respiratory symptoms: children treated as malaria, COVID-19 only considered in adults	Presumptive symptomatic treatment Hospital only when self-medication failed High malaria morbidity and complications
Malaria treatment	Focus on COVID-19, neglect of other diseases Shortage of antimalarials in rural areas	Patients handled as COVID-19 without confirmation Lack of antimalarials and limited opening of clinics leading to alternative medicine use

*ITN* insecticide treated net

### Theme 1: General effects of the COVID-19 pandemic

Sub-themes were identified as (a) finances, livelihood and food security, (b) health service provision, and (c) education and hygiene (Table 1). Direct impacts of the pandemic were limited, little or no COVID-19 infections were detected in most of the participating health facilities, and the mothers reported few to no COVID-19 cases in their communities. However, the indirect effects on life in general, on health care and malaria services were strongly felt for both mothers and HCPs, as shown by the following aspects.

Perceived as the strongest impact of the COVID-19 pandemic on mothers’ lives were the negative effects on jobs and increased financial difficulties. Restrictions in movement and social gatherings impeded the informal work sector, affecting mostly women and subsequently their children. The majority of them had insufficient personal income, which increased their dependency on their husbands, who had also frequently lost their jobs. The pandemic led to price increases of many commodities and transportation, which further increased the economic hardships and difficulties to access health care. Some women were forced to take loans they could not repay. The financial disruptions caused by COVID-19 also affected food security for both, urban and rural mothers, as prices of agricultural products and food increased and led some to think about fleeing: *“We even thought of running away from Ghana because we could not sell our things. How do you get money to buy food?”* (mother of child 2–5y, urban). Especially in rural communities, some families had difficulties paying school fees, with the situation pronounced for families where mother-ward pairs were students. HCPs affirmed the increased financial challenges on patients, adding that those who had no health insurance suffered the most.

Similar to the mothers, the general effects of the COVID-19 pandemic on HCPs centred on their work situation. Some reported a workload decrease as patient numbers reduced, while others experienced an increase as schedules were changed with prolonged working hours. Disease control officers particularly reported an increased workload as they were responsible for the COVID-19 management, adding additional burden to the surveillance of non-COVID-19 diseases. *“I was not always here, I was doing contact tracing. So the surveillance activities [for non-COVID-19 diseases] have not been done.”* (disease control officer, urban).

Many HCPs faced stigmatisation from their families and the community, as they were perceived as COVID-19 transmission sources. Further, HCPs reported misinformation about the virus. *“People had misinformation about what it was, a lot of different information, that it was a virus that was programmed or designed to affect Africans.”* (nurse, urban).

The belief that the virus did not exist was common in the society and the vaccination uptake was limited. HCPs reported funds being redirected by the government and external partners to COVID-19 related activities and thus reducing the funding of other routine care services. *“I had an impression that there was lack of money, it affected us. We received less funds. The whole attention was for COVID. For COVID, we had everything that we needed but for the other health issues, we were lacking.”* (nurse, urban).

Low patronage of health facilities also led to financial challenges as internally-generated funds were reduced as shown by this quotation: *“The hospital is run by what we generate internally. During COVID, the numbers dropped, so we are not getting the IGF [internally generated funds] to run our normal day-to-day activities.”* (nurse, rural). Similar to the mothers, the HCPs faced financial challenges in paying for transportation, which impacted their outreach services, especially in rural settings.

Most HCPs mentioned a lack of personal protective equipment (PPE) in health facilities, especially for non-COVID-19 related activities. Additionally, the use of PPE for outreach activities was impeded due to stigma associated with their wearing, and this increased the risk of infection among HCPs. *“If I would have dressed in a PPE, the person wouldn’t have allowed me to pick that [COVID-19] sample because of the community’s stigmatisation. So I had to go like that and I ended up infecting myself.”* (disease control officer, urban). Mothers reported negative aspects about nose masks, such as buying them at high prices if they needed to enter public places, including health facilities or markets to sell their goods.

Mothers and HCPs mentioned health education on COVID-19 via radio or television channels as well as community health volunteers to have helped in managing the pandemic, reaching the communities and motivating patients to visit health facilities again. However, mothers and HCPs felt that the sensitization about other diseases that present with COVID-19 like symptoms and the importance of seeking professional care was neglected as shown by these two quotes: *“Sometimes you may start thinking [you or your child have] the corona they are talking about because we were told that when you get it, you will be coughing, so when you experienced that, you only think about corona.”* (mother of child 6–23 m, rural).

*"I think that the education, the sensitization was not enough. Because COVID-19 has similar symptoms. So they didn’t sensitise the people enough for them to know that, if you are getting malaria you can get this particular symptom, but it doesn’t mean that it’s COVID-19, it can be other diseases. So they should still come to health care services. But they rather concentrated on COVID-19 signs and symptoms.”* (disease control manager, urban).

Mothers and HCPs alike revealed positive effects on overall hygiene, including hand washing. *“I think corona has brought us some good in our health, because it has increased people’s awareness of personal and environmental hygiene.”* (mother of child 2-5y, urban) *“The preventive measures that were put in place, even though they were meant for COVID, but in a way, they affected other conditions too, because the handwashing, nose masks, social distancing, in a way, they were also preventing other conditions like diarrhoea.”* (nurse, rural) The decrease in communicable diseases due to COVID-19 control measures led to a perceived decrease in the workload of some HCPs.

### Theme 2: Effects of the COVID-19 pandemic on health-seeking

Sub-themes were identified as (a) fear of COVID-19 infection, (b) COVID-19 testing, (c) behaviour of health care providers, (d) access to clinics and HCPs, and (e) access to medicines (Table 2).

Perspectives expressed by both, HCPs and mothers, showed that fear of getting infected with COVID-19 while visiting health facilities was the most important effect of the pandemic on general health care and related health-seeking behaviours, and was pronounced among patients with comorbidities, the elderly and pregnant women. There was also hesitancy in sending children to school due to the fear of getting infected with COVID-19 and non-COVID-19 diseases. Staff shortages were seen as attendance to work decreased due to fear of infection. In the tertiary facility, high risk staff such as pregnant women and the elderly staff were advised to stay at home and the antenatal care (ANC) unit provided limited services only on fixed appointments instead of open walk-in for some weeks.

Mothers also reported the lack of frontline HCPs, facility closures or reduced opening hours, especially for emergency services at night. *“There was a night that my child had a severe fever. We went to the hospital and it was locked. We had to knock on an over-the-counter owner to sell us a drug to ease it. It was a terrible night at the time of coronavirus.”* (mother of child 2–5y, rural).

Reports of not being palpated and weighed at the ANC led many women to stop seeking for those services. The consequences were home deliveries and reliance on traditional birth attendants with its associated birth complications. Attendance rate for routine health care services and childhood vaccinations dropped drastically. The ‘stay-at-home if your condition is less serious’ advice at the first peak of the COVID-19 pandemic contributed to reduced attendance numbers. Health facilities were only a last resort for patients when their home treatment failed or the condition worsened. “*So they [the patients] were out, they did not seek health care unless it’s an emergency case.”* (nurse, rural). Routine community-based child vaccinations were disrupted as many mothers stopped bringing their children to the clinics.

Beside the fear of infection and stigma when presenting with COVID-19 like symptoms, fear of testing positive and being isolated led many mothers to change their health-seeking behaviour and to manage the illness at home until the symptoms became severe: “*When you are not well, the fear of being tested positive would either delay or prevent you from seeing a doctor in a hospital.”* (mother of child 6–23 m, rural) This was also confirmed by HCPs: *“Most people were expecting that if you come to the hospital with fever, cough, or some of those symptoms, you would be classified as COVID, so people did not want to seek health care.”* (nurse, urban).

Many HCPs reported the possibilities for COVID-19 testing were very scarce or non-existent, as the antigen rapid diagnostic tests were only available in the tertiary centre through international research project supplies. As a result, suspected cases were not followed up in most facilities, leading to basing diagnosis and isolation decisions of suspected cases on presented symptoms.

Mothers opined that the behaviour of HCPs concerning patient care greatly affected their health-seeking behaviour. Many mothers alleged the non-availability of staff in health centres to provide treatment while some refused contact with symptomatic persons, especially those coughing. “*This child was sick and when we came [to the health centre], they refused to attend to him because of the high temperature he had.”* (mother of child 2–5y, urban) HCPs confirmed a change in treating their patients: *“As a health worker, you were afraid of a patient, you differently gave the patient care, so the care was compromised a bit.”* (nurse, rural) But also HCPs observed behavioural change among their patients, mainly due to patients’ fear of health staff infecting them and many HCPs reported stigmatisation from their families and the community: *“The cooperation of patients with the health care staff was also less because they see the staff as in higher risk [for COVID-19 infection], and it’s true.”* (nurse, rural) Social distancing also brought some positive changes for patients’ care, as the number of patients attended in one consulting room reduced leading to improved patient privacy.

Views of HCPs regarding medicine stocks in health facilities were quite varying. Some reported no effects on medicine stocks during the pandemic as the number of patients and subsequently the need for medications in health facilities reduced. However, some indicated shortages attributed to the pandemic or to general supply factors. *“In terms of the supplies, we still don’t have them out of COVID, so I’m not able to say if it was due to COVID or if this is just one of our challenges.”* (nurse, rural) In contrast, mothers experienced shortages in hospitals’ medication stocks. They had to buy the medicines that were not available in facilities at higher prices from privately-owned pharmacies and over-the-counter chemical shops. *“They will tell you there is no medicine due to corona so they will just write the medicine for you if you can afford and if you can’t afford it, this will affect you and the baby inside.”* (mother of child 6–23 m, urban) The need to pay for medication further increased financial challenges, adding to the already difficult financial situation of families: *“Here were times when your child was sick and the same amount [of farm products]that your husband earned [prior to the pandemic] to get you money to send your child to the hospital was not even enough to feed the family.”* (mother of child 2–5y, rural).

Over-the-counter drugs and herbal medications became the first option for symptom relief. *“The hospital was our last resort. You will give the child all the herbs you think would be of help, only when the condition became worse, we sent our children to the hospital. Especially if it involves cough and fever.”* (mother of child 2–5y, rural) However, some communities stopped using herbs from the bush as medications during the COVID-19 period because *“they said Corona was from bush animals. So those going to the bush to get these herbs, like the men in our house, were also afraid to go to the bush.”* (mother of child 2–5y, urban).

### Theme 3: Effects of the COVID-19 pandemic on malaria services

Sub-themes were identified as (a) malaria preventive measures, (b) differential diagnosis of malaria and COVID-19, and (c) malaria treatment (Table 3). Beside the general health service challenges, both rural and urban participants expressed concerns about the effects of COVID-19 on malaria-centred services. Key impacts were again financial deficits, following reallocations of funds by the government and international donors to ease COVID-19.

Malaria prevention programmes suffered some drawbacks. Medication for intermittent preventive treatment in pregnancy was out of stock in some facilities, especially in the rural communities, as well as ITNs. *“At the time of COVID, they will write for you to go and buy the [chemoprophylaxis] drugs and [the HCPs] will also not give you the net.”* (mother of child 6–23 m, rural) Some side effects of the chemoprophylaxis mimic COVID-19 symptoms, which reduced women’s willingness for drug uptake as shown by this quote: *“[…] when those pregnant women take SP [Sulfadoxine/Pyrimethamine], it gives them signs of COVID, temperature and other things. So because of that, they will not even take it.”* (nurse, urban).

Meanwhile, at the peak of the pandemic, when ANC services were limited, the time interval between the chemoprophylaxis distribution was inconsistent and extended. In most of the study communities, the seasonal malaria chemoprevention programmes for children under 5 years continued during the COVID-19 period leading to reductions in malaria morbidity. *“[Malaria] reduced, because the drugs that were given reduced the effect of malaria. Even when the child gets malaria, it does not worsen and then gets better again.”* (mother of child 2–5y, rural) The drug administration approach was changed from central distribution points to door-to-door administration by community nurses who also provided general health education. However, a challenge with the new approach was the stigmatisation of HCPs who entered the communities to provide this intervention as they were seen as possible sources of COVID-19 infection. Additionally, there was a lack of PPE for these interventions thereby increasing the risk of COVID-19 infection. *“We didn’t really have PPEs, we just went into the community and hoped that nothing bad happens.”*(nurse, urban).

Health care-seeking behaviour for malaria-related symptoms depended largely on general health education as malaria and COVID-19 share commonalities in symptoms: *“Education and sensitization were not enough because COVID-19 and malaria share some common symptoms like fever. […] if you are getting malaria, you can get this particular symptom, but it doesn’t mean that it’s COVID-19,[…]. So they should go to the health facilities for a sample to be taken.”* (disease control officer, urban).

Health facilities also provide education on common diseases such as malaria, but this has been missed by many patients during the pandemic: *“Malaria is one of the common things we educate on [at health facilities]. So, when you don’t come to seek health care, you don’t have the opportunity to be educated.”* (nurse, rural).

Hospitals became the last resort for severe malaria cases as patients stopped seeking professional help for mild symptoms, and this situation increased malaria fatality: *“From the statistics, we recorded quite a high number of [malaria] mortality, due to the fact that there is late attendance.” (nurse, rural).*

Except for the tertiary facility, there was no avenue to test for COVID-19. HCPs reportedly diagnosed COVID-19 using checklists based on clinical signs and symptoms in adults. Beside, some HCPs used malaria rapid diagnostic tests (RDTs) on the premise that a positive malaria test excludes a possible COVID-19 infection, neglecting the possibility of co-infections: *“What we also did were malaria test kits. When it tested positive, you can equally say that this patient has malaria and no COVID.”* (nurse, rural). Meanwhile, in the tertiary facility, cases of severe malaria and COVID-19 co-infection were documented. Among children presenting with general symptoms like fever, some HCPs reported initiating malaria treatment right away. Whereas other HCPs reported that during the peak of COVID-19, there were more malaria tests conducted than before and presumptive treatment based on malaria symptoms became less common. Some facilities reported malaria RDTs shortages during the COVID-19 peak. This compelled them to use microscopy blood smears which were time consuming and not available in rural facilities.

Overlapping of COVID-19 and malaria symptoms led to difficulties and misconceptions with patient management as there was extra focus on COVID-19. *“When my child had fever, we just knew that it was malaria but they treated us like it was corona, so they didn’t want to come closer to us.”* (mother of child 2–5y, urban) “*It was difficult at a point to diagnose, because the focus then was on COVID-19 and some of the symptoms mimic that of malaria. We were sometimes kind of overlooking the other diseases we used to treat and now focusing more on COVID-19.*” (medical doctor, tertiary facility, urban).

Mothers and some HCPs reported shortages in anti-malarials. Especially for simple malaria, presumptive symptomatic treatment by self-medication from over-the-counter stores or left-over orthodox and traditional medicine was the norm as shown by this quote: *“We were told not to seek health services from unlicensed practitioners and herbalists, but we had no choice at the time of the pandemic.”* (mother of child 6-23 m, rural).

In particular, the limited opening hours of health facilities due to COVID-19 social restrictions presented serious challenges for the malaria treatment, especially in the rural settings where the few health facilities are widely scattered.

## Discussion

This study tried to explain the quantitative changes in malaria cases seen in health facilities in northern Ghana in 2020 compared to 2015–2019 [20] by presenting health care professionals’ and mothers’ views on three different domains: effects on life in general, on health care and on malaria. The observed reductions in overall OPD visits as well as in malaria inpatient and OPD visits in 2020 compared to 2015–2019, that were strongest in inpatients and children under the age of five, could be explained by multidimensional effects on socio-economic inequality, access to and provision of health care. Health care professionals suffered stigmatisation, lacked funding and PPEs, while mothers had serious financial challenges, making them more dependent on their male counterparts. This aspect comes along findings of a study by Morgan et al. [29]: Women constitute a majority of frontline HCPs, exposing them more to COVID-19 infections, women suffered more from sexual violence during the pandemic and girls in LMICs were at high risk to get into forced marriage due to school closures, an aspect that was also mentioned during informal interviews at the study site.

Many women also mentioned the increase in food insecurity as one of the consequences of the COVID-9 pandemic. Women represent two thirds of farm labourers in SSA and the increase in food instability has further increased gender inequity [30]. COVID-19 has once more highlighted the importance of stress-resistant public health structures, frontline HCPs and accessible and functioning health care systems. Frontline HCPs and community health care workers play a crucial role in malaria elimination; they work directly with affected communities and provide general health care services and health education even when health facilities become inaccessible as experienced during the pandemic. A study in Rwanda found a decrease in malaria RDTs conducted in facilities and an increase in RDTs conducted in communities, highlighting the need to enter communities during crises; the test positivity rate for malaria increased during the pandemic, emphasising the increase in morbidity. Further, similar to our study, they found changes in the behaviour of HCPs and difficulties with communities to reach health facilities due to lack of transportation. Also, education on non-COVID-19 diseases was reduced [31]. A qualitative study by Taremwa et al. [32] in Uganda generated similar findings, emphasising the negative effects of the pandemic on malaria by a reduced access to health care and disrupted prevention activities. The pandemic compromised health care through financial challenges, fear as well as difficulties in the clinical handling of COVID-19 alongside malaria and the associated increase in socio-economic inequities as supported by a qualitative study with focus on malaria mass testing, treatment and tracing in a rural community in the Eastern Region of Ghana. Overlapping symptoms of malaria and COVID-19 reduced the health facility attendance and changed the treatment behaviour of HCPs, HCPs were stigmatised as infection sources, and misinformation about COVID-19 vaccinations and testing affected the participation in malaria prevention programmes. [21] Amu et al. [33] set the focus in their research on health system functioning in SSA, supporting the result of limited financial resources in health facilities, leading to following result: “…the majority of the population in SSA still suffer financial barriers as out-of-pocket expenditure is required before essential medical care can be delivered, even in emergencies. In such situations, the most vulnerable (poor), therefore, bear the highest burden of diseases and high levels of health expenditure. The already fragile health systems are overburdened with the grave task to address the COVID-19 pandemic.” [33].

Another collateral damage is the increase in adverse maternal events: more pregnant women avoided ANC and skilled birth attendants. The previous data from northern Ghana showed increases in malaria morbidity in pregnant women in 2020 [20]. This trend was in contrast to the dynamics in all the other patient groups. Hypotheses explaining this were disrupted ANC services including malaria preventive activities as chemoprophylaxis during pregnancy and ITN distribution. Without those public health interventions, more pregnant women were at risk for malaria infection; a hypothesis which was supported by the findings from our qualitative study.

This study has strengths and limitations: one major limitation is the missing age information of the study participants. This information was not retrieved as the focus was on the children’s age or the pregnancy period of the women. Every participant was only asked if she/he is over 18 years old. Nevertheless, the exact age would have been helpful to judge the individual experience of the participants. However, one major strength is the large number of participants, including various perspectives of the different communities including rural and urban experiences, and mentioning HCPs’ and mothers’views on the same topic.

## Conclusion

Fortunately, worst case scenarios with large increases of the malaria burden in SSA as modelled at the onset of the COVID-19 pandemic were averted following a rapid adaptation of local health structures. However, this study shows the various negative effects the pandemic had on everyday life, health care and the malaria situation for mothers and HCPs, especially the aggravation of social inequity is a global challenge. More research focusing on the control and elimination of diseases related to poverty (e.g. malaria) is needed to fully understand the consequences of the pandemic on vulnerable populations.

## Data Availability

The data used and/or analysed during the current study are available from the corresponding author on reasonable request.
